# Retraction: miR-221/222 promote tumor growth and suppress apoptosis by targeting lncRNA GAS5 in breast cancer

**DOI:** 10.1042/BSR-20181859_RET

**Published:** 2021-04-19

**Keywords:** apoptosis, breast cancer, GAS5, miR-221/222

This article is being retracted from *Bioscience Reports* at the request of the Editorial Board following receipt of a notification from a reader, alerting the Editorial Office to very similar data plots in Figure 4A.

The authors contacted the Editorial Office, providing a revised Figure 4, explaining that the similarity may be due to the same sample being re-tested by mistake. However, as the original data has been lost and there are concerns surrounding the sample and data storage, the Editorial Board stand by the decision to retract the article. The authors disagree to the retraction.

